
JOURNAL INFORMATION
==============================
NLM Title Abbreviation: ScientificWorldJournal
Journal ID: ScientificWorldJournal
NLM Journal ID: 101131163
Journal ID: TSWJ

The Scientific World Journal

ISSN: 2356-6140
EISSN: 1537-744X
Publisher: Wiley

ARTICLE INFORMATION
==============================
PMCID: PMC5849075
PMID: 18979052
DOI: 10.1100/tsw.2008.145
Article ID: 348056
Article version: 1
Subjects: Directions in Science

Vascular Pathology and the Role of Hyaluronan

Vigetti Davide
Viola Manuela
Karousou Evgenia
Genasetti Anna
Rizzi Manuela
Clerici Moira
Bartolini Barbara
Moretto Paola
De Luca Giancarlo
Passi Alberto *
Department of Experimental Biomedical and Clinical Sciences (DSBSC), University of Insubria, Varese, Italy
*Alberto Passi: alberto.passi@uninsubria.itAcademic Editor: Martin Goette

Publication date: 2008
Electronic publication date: 2008 Nov 2
Volume: 8
First page: 1116
Last page: 1118
Received 2008 Jul 30; Revised 2008 Oct 6; Accepted 2008 Oct 10
Copyright: Copyright © 2008 Davide Vigetti et al.
Copyright year: 2008
License: This is an open access article distributed under the Creative Commons Attribution License, which permits unrestricted use, distribution, and reproduction in any medium, provided the original work is properly cited.
License URL: https://creativecommons.org/licenses/by/3.0/

Keywords: glycosaminoglycan, aortic smooth muscle cells, aging, cell migration, CD44, erk1/2, restenosis

==============================
Directions in Science
*TheScientificWorldJOURNAL* (2008) 8, 1116–1118
ISSN 1537-744X; DOI 10.1100/tsw.2008.145

TheScientificWorldJOURNAL
www.thescientificworld.com

### Vascular Pathology and the Role of Hyaluronan

Davide Vigetti, Manuela Viola, Evgenia Karousou, Anna Genasetti, Manuela Rizzi, Moira Clerici, Barbara Bartolini, Paola Moretto, Giancarlo De Luca, and Alberto Passi*

*Department of Experimental Biomedical and Clinical Sciences (DSBSC), University of Insubria, Varese, Italy*

**E-mail:** davide.vigetti@uninsubria.it; Manuela.viola@uninsubria.it, jenny.karousou@uninsubria.it, anna.genasetti@uninsubria.it, manuela.rizzi@uninsubira.it, moira.clerici@uninsubria.it, barbara.bertolini@uninsubria.it, paola.moretto@uninsubria.it, giancarlo.deluca@uninsubria.it; alberto.passi@uninsubria.it

*Received July 30, 2008; Revised October 6, 2008; Accepted October 10, 2008; Published November 2, 2008*

**KEYWORDS**: glycosaminoglycan, aortic smooth muscle cells, aging, cell migration, CD44, erk1/2, restenosis

The major cause of death in humans is directly related to vascular diseases[1]. The development of vascular pathology is often coupled to dramatic alterations of the extracellular matrix (ECM), which provides critical support for vascular tissue as a scaffold for maintaining the organization of vascular cells into blood vessels, for blood vessel stabilization, morphogenesis, and for cell proliferation, migration, and survival[2]. The ECM constitutes a complex milieu of macromolecules that influence, throughout specific interactions, the activities of the cells, including cell migration and proliferation. Hyaluronan (HA) is an important component of the ECM that has generated increasing interest within the scientific community due to its multitude of functions. HA is a linear polymer belonging to the family of glycosaminoglycans (GAGs), which comprises the major fraction of carbohydrates in ECM[3] . Even though HA is not covalently bound to a protein core as the other GAGs that form proteoglycans, it can interact with several proteinaceous molecules (hyaloadherins)[4]. HA is a substrate onto which other proteins could bind, including proteoglycans that can assemble, forming a complex network in ECM. This aggregation maintains the physical form and the biochemical properties of tissues, underlining that HA has not only a structural function, but it also is a regulator of cellular behavior[5].

Cell motility is critical in several pathologies and in vascular biology, human aortic smooth muscle cell (AoSMC) migration represents a crucial point in the onset of pathology[6]. In normal vessels, AoSMCs are mainly located within the tunica media of blood vessels in a contractile state, expressing specific genes essential for cell function, such as regulation of vessel tone and blood pressure[7]. A large body of scientific evidence supports the concept that one of the most precocious events during the onset of arteriosclerosis or restenosis in vessels is the AoSMC migration towards the intima layer. In fact, under pathological conditions, such as vessel injury or arteriosclerotic plaque development, AoSMCs become exposed to certain growth factors, inflammatory cytokines, and other circulating components that induce a transformation from the contractile to a synthetic state[8]. Only in this synthetic behavior do the AoSMCs acquire the ability to proliferate, migrate, and accumulate within the intimal layer of arteries

*Corresponding author.
©2008 with author.
Published by TheScientificWorld; www.thescientificworld.com under the influence of chemotaxis-inducing growth factors. During the migration, AoSMCs have been shown to digest major extracellular barriers, such as basal membranes, interstitial collagens, and proteoglycans[9].

It is well known that the onset of vascular pathologies increases with aging and, interestingly, even AoSMCs change their gene expression pattern and their ECM composition during aging, exerting direct effects on various cellular events and cell migration[10]. In fact, a role for HA during AoSMC migration was recently established and, therefore, its possible involvement in vascular pathology[10]. In recent papers, it was demonstrated that the AoSMCs produce large amounts of HA[11] and that the ECM strongly influences cell migration[12]. The data obtained on HA synthesis in AoSMCs during aging confirm the idea that the ECM modification can alter cell migration. *In vitro*, aged cells produced more HA and its receptor CD44 on the cell membrane. In this *in vitro* aging model, primary AoSMCs were cultured for several population doublings and cells with a high number of mitosis were considered aged. The robustness of this study was achieved by measuring several markers of senescence (as P16 and galactosidase activity) and verifying the phenotype of cells during the senescence process *in vitro*. The role of HA and its receptor CD44 on AoSMC motility was demonstrated as blocking CD44 activity. In fact, experiments that carried out blocking the receptor or its signal transmission (the ERK1/2 phosphorylation) abolished the HA effect on cell motility[10]. Moreover, modifying the HA content in the cells by adding HA of different molecular size to the cell culture at different concentrations increased cell motility. In fact, the HA size is critical for this modulation as low-molecular-weight HA does not show the same effect observed using high-molecular-weight HA. However, as other pathways involving VCAM and ICAM proteins could potentially control the migratory capability of AoSMCs, the involvement of VCAM and ICAM throughout the evaluation of the expression in young and aged AoSMCs of these molecules was ruled out. Therefore, HA, through CD44 and ERK phosphorylation, was shown to control AoSMC migration. These experiments demonstrate that the interaction of HA and cell receptor CD44 is critical in the AoSMC motility, highlighting the role of the microenvironment in cell migration[10,11,12].

In the literature, it was shown that the amount of HA increases during the early arteriosclerotic process, indicating that this polymer could play a critical role in this process[13]. In the model of aged AoSMCs, it was demonstrated that HA production, as well as the synthesis of its receptor CD44[9], increased and that the accumulation of HA in AoSMCs is specific, as the other GAGs did not increase their amount during *in vitro* aging. The analysis of AoSMC cell motility in aged cells demonstrated that the increased HA induces a higher motility and that this phenomenon was CD44 dependent. It is known that CD44 promotes arteriosclerosis[14] and that AoSMC migration during aging depends on HA, CD44, and the phosphorylation of MAP kinase ERK 1/2. Because CD44 signaling can modify the activity of the extracellular signal-related kinase ERK 1/2[15], the high CD44 expression in aged cells could mediate higher levels of phosphorylated ERK1/2 protein. Hence, CD44 and RHAMM (another HA receptor) can influence AoSMC behavior[16,17]; the expression of CD44 was significantly increased in both aged AoSMCs as well as in adult human aorta RNA[9], supporting the idea that the *in vitro* cell model of AoSMCs resembles an *in vivo* condition[10]. It was reported that CD44-modulating cell motility could play a role in several pathologies[18].

Considering the role of HA in the AoSMC migration, it may be reasonable to assume that the inhibition of HA synthesis *in vivo* in the vascular tissue may contribute to reduction of the cell migration. 4-Metyllumbelliferone (4-MU), a well-known HA synthesis inhibitor[19], could be considered as a candidate for drug-eluting stents in order to prevent cell migration and, in final extent, the vessel restenosis. Moreover, other approaches are available in therapy to inhibit restenosis, as drug-eluting stents that release sirolimus or paclitaxel that arrest SMC cell cycle and migration[20]. Recently, sirolimus has been discovered to inhibit vascular SMC HA secretion[21] and the role of hyaluronan synthases expression was related to the monocyte adhesion in SMC[22]. By reducing HA synthesis and therefore the AoSMC motility, 4-MU could represent a new molecule with additional beneficial pharmacological effects *in vivo*.

### REFERENCES

1. Mathers, C.D., Fat, D.M., Inoue, M., Rao, C., and Lopez, A.D. (2005) Counting the dead and what they died from: an assessment of the global status of cause of death data. *Bull. World Health Organ.* **83**, 171–177.
2. Davis, G.E. and Senger, D.R. (2005) Endothelial extracellular matrix: biosynthesis, remodeling, and functions during vascular morphogenesis and neovessel stabilization. *Circ. Res.* **97**, 1093–1107.
3. Karamanos, N.K., Axelsson, S., Vanky, P., Tzanakakis, G.N., and Hjerpe, A. (1995) Determination of hyaluronan and galactosaminoglycan disaccharides by high-performance capillary electrophoresis at the attomole level. Applications to analyses of tissue and cell culture proteoglycans. *J. Chromatogr. A* **696**, 295–305.
4. Spicer, A.P. and Tien, J.Y. (2004) Hyaluronan and morphogenesis. *Birth Defects Res. C Embryo Today* **72,** 89–108.
5. Day, A.J. and Prestwich, G.D. (2002) Hyaluronan-binding proteins: tying up the giant. *J. Biol. Chem.* **277**, 4585–4588.
6. Wight, T.N. (2008) Arterial remodeling in vascular disease: a key role for hyaluronan and versican. *Front. Biosci.* **13**, 4933–4937.
7. Ross, R. (1993) The pathogenesis of atherosclerosis: a perspective for the 1990s. *Nature* **362,** 801–809.
8. Halayko, A. J. and Solway, J. (2001) Molecular mechanisms of phenotypic plasticity in smooth muscle cells. *J. Appl. Physiol.* **90**, 358–368.
9. Passi, A., Negrini, D., Albertini, R., Miserocchi, G., and De Luca, G. (1999) The sensitivity of versican from rabbit lung to gelatinase A (MMP-2) and B (MMP-9) and its involvement in the development of hydraulic lung edema. *FEBS Lett.* **456,** 93–96.
10. Vigetti, D., Viola, M., Karousou, E., Rizzi, M., Moretto, P., Genasetti, A., Clerici, M., Hascall, V.C., De Luca, G., and Passi, A. (2008) Hyaluronan-CD44-erk1/2 regulate human aortic smooth muscle cell motility during aging. *J. Biol. Chem.* **283**, 4448–4458.
11. Vigetti, D., Ori, M., Viola, M., Genasetti, A., Karousou, E., Rizzi, M., Pallotti, F., Nardi, I., Hascall, VC., De Luca, G., and Passi, A. (2006) Molecular cloning and characterization of UDP-glucose dehydrogenase from the amphibian Xenopus laevis and its involvement in hyaluronan synthesis. *J. Biol. Chem.* **281**, 8254–8263.
12. Vigetti, D., Moretto, P., Viola, M., Genasetti, A., Rizzi, M., Karousou, E., Pallotti, F., De Luca, G., and Passi, A.(2006) Matrix metalloproteinase 2 and tissue inhibitors of metalloproteinases regulate human aortic smooth muscle cell migration during in vitro aging. *FASEB J.* **20(8)**, 1118–1130.
13. Riessen, R., Wight, T.N., Pastore, C., Henley, C., and Isner, J.M. (1996) Distribution of hyaluronan during extracellular matrix remodeling in human restenotic arteries and balloon-injured rat carotid arteries. *Circulation* **93**, 1141–1147.
14. Cuff, C.A., Kothapalli, D., Azonobi, I., Chun, S., Zhang, Y., Belkin, R., Yeh, C., Secreto, A., Assoian, R.K., Rader, D.J., and Pure, E. (2001) The adhesion receptor CD44 promotes atherosclerosis by mediating inflammatory cell recruitment and vascular cell activation. *J. Clin. Invest.* **108**, 1031–1040.
15. Toole, B.P. (2004) Hyaluronan: from extracellular glue to pericellular cue. *Nat. Rev. Cancer* **4,** 528–539.
16. Savani, R.C., Wang, C., Yang, B., Zhang, S., Kinsella, M.G., Wight, T.N., Stern, R., Nance, D.M., and Turley, E.A. (1995) Migration of bovine aortic smooth muscle cells after wounding injury. The role of hyaluronan and RHAMM. *J. Clin. Invest.* **95**, 1158–1168.
17. Gouëffic, Y., Guilluy, C., Guérin, P., Patra, P., Pacaud, P., and Loirand, G. (2006) Hyaluronan induces vascular smooth muscle cell migration through RHAMM-mediated PI3K-dependent Rac activation. *Cardiovasc. Res.* **72**, 339–344.
18. Isacke, C.M. and Yarwood, H. (2002) The hyaluronan receptor, CD44. *Int. J. Biochem. Cell Biol.* **34**, 718–721.
19. Nakamura, T., Takagaki, K., Shibata, S., Tanaka, K., Higuchi, T. and Endo, M. (1995) Hyaluronic-acid-deficient extracellular matrix induced by addition of 4-methylumbelliferone to the medium of cultured human skin fibroblasts. *Biochem. Biophys. Res. Commun.* **208**, 470–475.
20. Parry, T.J., Brosius, R., Thyagarajan, R., Carter, D., Argentieri, D., Falotico, R., and Siekierka, J. (2005) Drug-eluting stents: sirolimus and paclitaxel differentially affect cultured cells and injured arteries. *Eur. J. Pharmacol.* **524**, 19–29.
21. Gouëffic, Y., Potter-Perigo, S., Chan, C.K., Johnson, P.Y., Braun, K., Evanko, S.P., and Wight, T.N. (2007) Sirolimus blocks the accumulation of hyaluronan (HA) by arterial smooth muscle cells and reduces monocyte adhesion to the ECM. *Atherosclerosis* **195**, 23–30.
22. Wilkinson, T.S., Bressler, S.L., Evanko, S.P., Braun, K.R., and Wight, T.N. (2006) Overexpression of hyaluronan synthases alters vascular smooth muscle cell phenotype and promotes monocyte adhesion. *J. Cell. Physiol.* **206(2),** 378–385.

**This article should be cited as follows:**

Vigetti, D., Viola, M., Karousou, E., Genasetti, A., Rizzi, M., Clerici, M., Bartolini, B., Moretto, P., De Luca, G., and Passi, A. (2008) Vascular pathology and the role of hyaluronan. *TheScientificWorldJOURNAL* **8**, 1116–1118. DOI 10.1100/tsw.2008.145.
